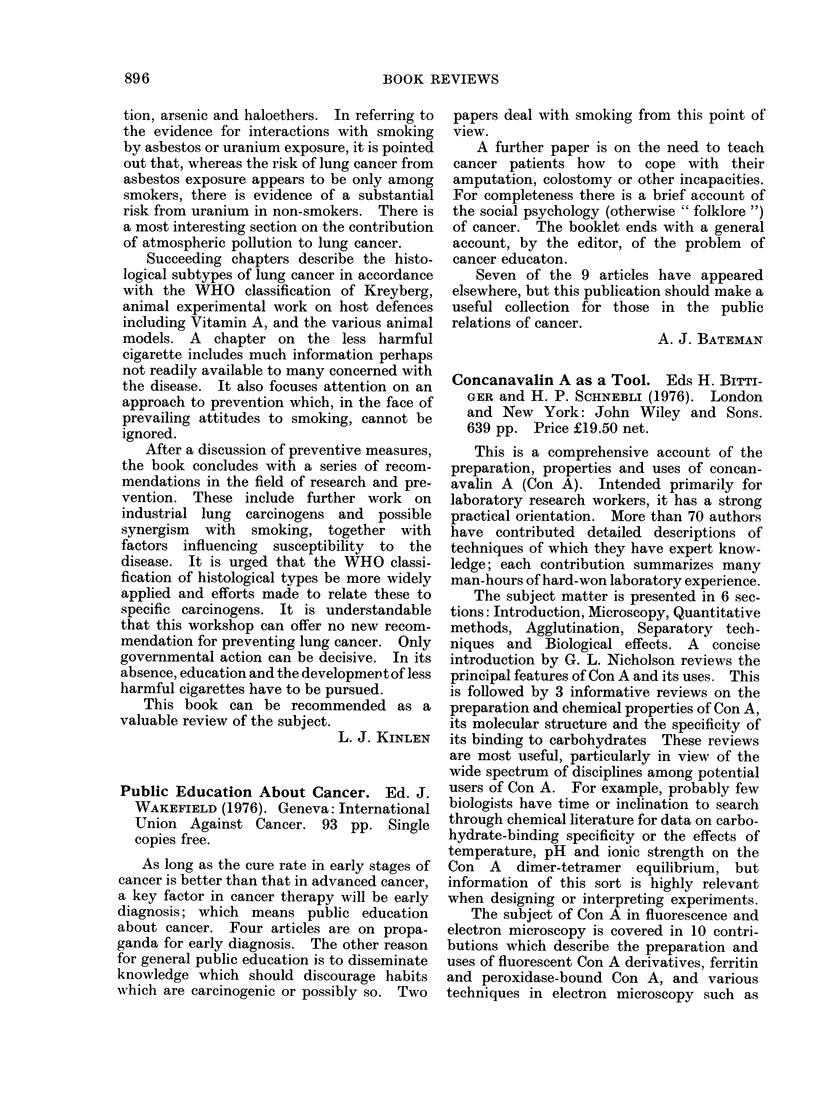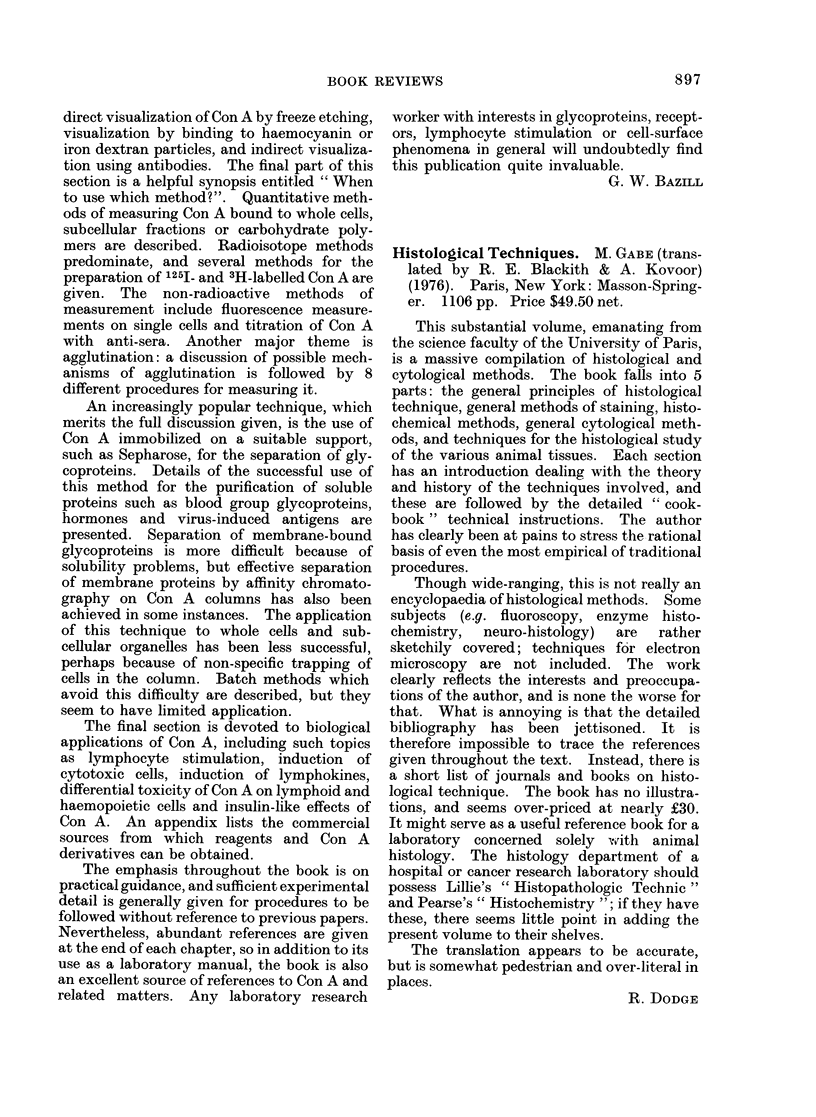# Concanavalin A as a Tool

**Published:** 1977-06

**Authors:** G. W. Bazill


**Concanavalin A as a Tool.** Eds H. BITTIGER and H. P. SCHNEBLI (1976). London and New York: John Wiley and Sons. 639 pp. Price £19.50 net.

This is a comprehensive account of the preparation, properties and uses of concanavalin A (Con A). Intended primarily for laboratory research workers, it has a strong practical orientation. More than 70 authors have contributed detailed descriptions of techniques of which they have expert knowledge; each contribution summarizes many man-hours of hard-won laboratory experience.

The subject matter is presented in 6 sections: Introduction, Microscopy, Quantitative methods, Agglutination, Separatory techniques and Biological effects. A concise introduction by G. L. Nicholson reviews the principal features of Con A and its uses. This is followed by 3 informative reviews on the preparation and chemical properties of Con A, its molecular structure and the specificity of its binding to carbohydrates These reviews are most useful, particularly in view of the wide spectrum of disciplines among potential users of Con A. For example, probably few biologists have time or inclination to search through chemical literature for data on carbohydrate-binding specificity or the effects of temperature, pH and ionic strength on the Con A dimer-tetramer equilibrium, but information of this sort is highly relevant when designing or interpreting experiments.

The subject of Con A in fluorescence and electron microscopy is covered in 10 contributions which describe the preparation and uses of fluorescent Con A derivatives, ferritin and peroxidase-bound Con A, and various techniques in electron microscopy such as direct visualization of Con A by freeze etching, visualization by binding to haemocyanin or iron dextran particles, and indirect visualization using antibodies. The final part of this section is a helpful synopsis entitled " When to use which method?". Quantitative methods of measuring Con A bound to whole cells, subcellular fractions or carbohydrate polymers are described. Radioisotope methods predominate, and several methods for the preparation of $^{125}$I- and $^{3}$H-labelled Con A are given. The non-radioactive methods of measurement include fluorescence measurements on single cells and titration of Con A with anti-sera. Another major theme is agglutination: a discussion of possible mechanisms of agglutination is followed by 8 different procedures for measuring it.

An increasingly popular technique, which merits the full discussion given, is the use of Con A immobilized on a suitable support, such as Sepharose, for the separation of glycoproteins. Details of the successful use of this method for the purification of soluble proteins such as blood group glycoproteins, hormones and virus-induced antigens are presented. Separation of membrane-bound glycoproteins is more difficult because of solubility problems, but effective separation of membrane proteins by affinity chromatography on Con A columns has also been achieved in some instances. The application of this technique to whole cells and subcellular organelles has been less successful, perhaps because of non-specific trapping of cells in the column. Batch methods which avoid this difficulty are described, but they seem to have limited application.

The final section is devoted to biological applications of Con A, including such topics as lymphocyte stimulation, induction of cytotoxic cells, induction of lymphokines, differential toxicity of Con A on lymphoid and haemopoietic cells and insulin-like effects of Con A. An appendix lists the commercial sources from which reagents and Con A derivatives can be obtained.

The emphasis throughout the book is on practical guidance, and sufficient experimental detail is generally given for procedures to be followed without reference to previous papers. Nevertheless, abundant references are given at the end of each chapter, so in addition to its use as a laboratory manual, the book is also an excellent source of references to Con A and related matters. Any laboratory research worker with interests in glycoproteins, receptors, lymphocyte stimulation or cell-surface phenomena in general will undoubtedly find this publication quite invaluable.

G. W. Bazill